# Speed up discharge planning at the acute stroke unit: A development and external validation study for the early prediction of discharge home

**DOI:** 10.3389/fneur.2022.999595

**Published:** 2022-09-16

**Authors:** Janne Marieke Veerbeek, Beatrice Ottiger, Dario Cazzoli, Tim Vanbellingen, Thomas Nyffeler

**Affiliations:** ^1^Neurocenter, Luzerner Kantonsspital, Lucerne, Switzerland; ^2^ARTORG Center for Biomedical Engineering Research Gerontechnology and Rehabilitation Group, University of Bern, Bern, Switzerland; ^3^Department of Psychology, University of Bern, Bern, Switzerland; ^4^Department of Neurology, Inselspital, Bern University Hospital, University of Bern, Bern, Switzerland

**Keywords:** stroke, stroke unit, prediction, discharge, validation, decision tree, cohort study

## Abstract

**Background:**

To reduce healthcare costs, it has become increasingly important to shorten the length of stay in acute stroke units. The goal of this study was to develop and externally validate a decision tree model applicable < 48 h poststroke for discharge home from an acute stroke unit with a short length of stay, and to assess the inappropriate home discharge rate.

**Methods:**

A prospective study including two samples of stroke patients admitted to an acute stroke unit. The outcome was discharge home (yes/no). A classification and regression tree analysis was performed in Sample 1. The model's performance was tested in Sample 2.

**Results:**

In total, 953 patients were included. The final decision tree included the patients' activities of daily living (ADL) performance <48 h poststroke, including motor function, cognition, and communication, and had an area under the curve (AUC) of 0.84 (95% confidence interval 0.76, 0.91). External validation resulted in an AUC of 0.74 (95% confidence interval 0.72, 0.77). None of the patients discharged home were re-admitted < 2 months after discharge to a hospital or admitted to a rehabilitation center for symptoms that had needed inpatient neurorehabilitation.

**Conclusions:**

The developed decision tree shows acceptable external validity in predicting discharge home in a heterogeneous sample of stroke patients, only based on the patient's actual ADL performance <48 h poststroke. Importantly, discharge was safe, i.e., no re-hospitalization was registered. The tree's application to speed up discharge planning should now be further evaluated.

## Introduction

A quick start of discharge planning for patients admitted to an acute hospital stroke unit is essential in order to reduce healthcare costs and to allow patients a trajectory aiming for an optimized outcome. Prediction models can be a useful tool to support objective decision-making regarding the patient's discharge destination. Different prediction models for the discharge destination from an acute stroke setting have been developed. Important predictors for discharge destination were found to be the patient's age, living situation prior to stroke, dependence in the activities of daily living (ADLs) after admission, including cognitive aspects thereof, and level of neurological deficits (1, 2). Although an external validation of prediction models in an independent, prospective sample of patients is an essential step toward their clinical application, externally validated models are however scarce. The importance of external validation is supported by the observation that the application of a prediction model in a sample of patients that was not involved in the development of the model itself most often results in poorer model performance (3).

Recently, Itaya et al. developed (4) and temporally validated (5) a model that predicts the likelihood of being discharged home in a sample of stroke patients who had a mean length of hospital stay of 23.6 ± 12.8 days in the development study and 17.2 ± 9.3 days in the temporal validation study. The prediction model included the patients' premorbid living situation, type of stroke, ADL independence at admission, and the presence of a paresis. Although the discriminative power [i.e., the area under the receiver operator characteristic curve (AUC)] of 0.88 and 0.80 in the development (4) and validation sample (5), respectively, was good, this Asian model is not directly applicable to hospital settings in which the length of stay is considerably shorter, such as in Switzerland [median 7.6 days (6)] or the United States [mean 5.3 days (7)]. As the length of hospital stay after a stroke has been constantly decreasing over time in order to reduce healthcare costs (7), this lack of model transferability will become a more global problem. The authors of the Itaya model (4) furthermore criticized the fact that they did not possess information concerning the rate of patients who were inappropriately discharged home. Getting insight into this rate is important, as early and inappropriate discharge home can result in adverse events, such as early hospital re-admissions [an important quality indicator for hospital care (8)], increasing healthcare costs (9), as well as negatively impacting on the patients' outcome and quality of life.

Therefore, our goal was to develop an easy-to-apply decision tree applicable already within 48 h after stroke for discharging patients safely home from an acute stroke unit setting, in the context of a Swiss hospital with a short length of stay (<1 week). The decision tree was externally validated in an independent, heterogeneous sample of patients that was recruited at a later time point (i.e., temporal external validation), to allow for a broad application spectrum of the developed decision tree. In addition, the inappropriate home discharge rate was evaluated. We hypothesized that the patient's actual ADL performance at the acute stroke unit would be essential for predicting the discharge destination, and that the temporal external validation would result in an acceptable decision tree performance.

## Materials and methods

### Data disclosure statement

The data that support the findings of this study are available from the corresponding author by qualified researchers upon reasonable request.

### Design

This work is based on data that were prospectively collected within clinical routine in acute stroke patients consecutively admitted to the Stroke Center of the Neurology Department of the Luzerner Kantonsspital, Lucerne, Switzerland. This hospital is the largest hospital in central Switzerland, with a catchment population of 800,000 inhabitants. The data are stored in a local registry. Sample 1 (i.e., the development cohort) includes data of patients who entered the hospital between April and November 2019. Sample 2 (i.e., the validation cohort) includes patient data collected between April 2020 and March 2022.

Ethical approval was obtained from the cantonal ethics committee Northwest and Central Switzerland (EKNZ; BASEC-ID 2017-00998). All patients signed the hospital's general consent form, which allows to analyze the data collected within clinical routine for research purposes. The work adhered to the World Medical Association Declaration of Helsinki (10) and reporting adhered to the STROBE (11) and TRIPOD statements (12).

### Participants

Both samples included patients who suffered an acute stroke. No in- or exclusion criteria (such as the number of strokes, stroke type, presence of comorbidities, or pre-stroke independence) were formulated. Patients received medical and rehabilitative treatment according to Swiss national guidelines (13). Occupational and physical therapy were problem- and task-oriented and had a repetitive nature.

Discharge destination was decided by the physician in charge in close collaboration with the other members of the multidisciplinary team and was discussed with the patient and/or caregivers. The recommendation was based on multiple factors, such as the patient's neurological impairments, ADL functioning, Montreal Cognitive Assessment, Functional Ambulation Categories, premorbid modified Rankin Scale, and premorbid living situation.

### Data collection and variables

#### Dependent variable

This study's dependent variable was discharge home (no/yes). Discharge home was defined as living at home with or without partner and/or family member(s), regardless of whether support was needed. Patients who were transferred into a rehabilitation center or a nursing home, another acute hospital or to temporal transitional care, or those who died during hospital stay were classified as not discharged home.

#### Independent variables

The following candidate predictors were considered, because these have been shown to be most predictive for discharge destination (1, 2, 4): age, sex, acute neurological deficits as assessed with the National Institutes of Health Stroke Scale (NIHSS) (14, 15) at hospital admission (or in the case of acute reperfusion therapy: after thrombolysis and/or thrombectomy), stroke type (ischemic/hemorrhagic), recurrent stroke (yes/no), living at home with or without support (yes/no), and the patient's actual performance in the ADLs at the acute stroke unit, including cognition and communication, as assessed with the short version of the Lucerne ICF-Based Multidisciplinary Observation Scale (Short-LIMOS) (16) < 48 h poststroke. In brief, the Short-LIMOS is a 10-item observation scale that includes motor, cognitive, and communication items and has a score range of 10–50, with higher scores indicating a better performance. The administration time of the Short-LIMOS is < 10 min, and the scale is described in more detail in the Supplementary material. Trained occupational and physiotherapists performed the Short-LIMOS observation during routine care in the pilot phase from 04–11/2019 and after definite implementation from 04/2020 onwards. The patients were observed by therapists within the previously mentioned 48-h time frame and the Short-LIMOS items were assessed once. In case acute reperfusion therapies were applied, the Short-LIMOS observation took place after application of these acute medical therapies. The other data relevant for this study were extracted from the electronic hospital information system.

#### Inappropriate discharge home

Additionally, inappropriate discharge home was defined as early readmission (within the first 2 months after hospital discharge) to any hospital or rehabilitation clinic because of stroke-related symptoms (pneumonia after dysphagia, unable to manage everyday life with or without support of caregivers or home-care services, etc.) that would have required inpatient neurorehabilitation. This information was collected as a quality measure by means of a telephone call with the patient or his/her caregiver.

#### Sample size

The number of patients included in the present study matches previous work in the field using classification and regression tree (CART) analyses (17–19). For external validation, a minimum number of events was set to 100 (20, 21).

### Statistical analysis methods

Patient characteristics of both samples were analyzed by means of descriptive statistics (medians with 1st and 3rd quartiles, or frequencies with percentages), and non-parametric tests were applied to determine differences between them (Mann-Whitney U test, Chi-squared test).

A CART analysis (22) was performed in Sample 1 (development sample) in order to develop a decision tree for discharge home (yes/no) and the above-mentioned independent variables (see Section Independent variables) were used without transformation or dichotomization. In this approach, the data on the predictors are partitioned to create homogeneous groups on the dependent categorical variable. The tree starts with the parent node and each split of the tree results in two subsequent (child) nodes. For each split of the tree, the CART analysis selects the best predictor with its cut-off that represents the highest improvement (i.e., the largest decrease) in the Gini index by subtracting the weighted mean of the two child nodes from the value of the node from which the two child nodes originate. The Gini index is a measure for impurity of a node, based on the dependent variable: the higher the value, the higher the impurity of that node (22, 23). This partitioning is repeated recursively until no further improvement can be made or until pre-defined stopping rules are met (e.g., no less than five patients in a node). In our case, first, a tree was built unrestrictedly, and internally validated using a 10-fold cross-validation. To prevent overfitting, this tree was subsequently pruned by selecting the lowest cross-validation error with its corresponding cost-complexity parameter, which is defined by the number of nodes in the tree and the error rate. An advantage of the CART analysis is that it is non-parametric, can deal with missing predictor data by using surrogate markers (i.e., taking the next-best splitting predictor with corresponding cut-off that has the next-best improvement in Gini index), and results in an easy-to-interpret decision tree. The surrogate marker is only taken in patients for which data on the predictor that was selected by the CART analysis was not available. This means that patients with missing data were not removed (i.e., not a complete-case analysis), which reduces bias (24) and, in addition, reflects clinical practice.

The decision tree's performance was assessed in terms of overall accuracy, sensitivity, specificity, positive and negative predictive values, and AUC. The AUC was classified as inappropriate (~0.50), acceptable (0.70 ≤ AUC < 0.80), excellent (0.80 ≤ AUC < 0.90), and outstanding (≥0.90) (25). Thereafter, the decision tree's performance was externally validated in Sample 2 (validation cohort). In Sample 2, differences in baseline characteristics between patients with and without missing predictor data of the developed decision tree were analyzed by non-parametric statistics.

Descriptive statistics were applied in order to investigate inappropriate discharge home.

R was used for all analyses (26) and the R package “rpart” (27) was used for the CART analysis. A *p*-value of <0.05 (two-tailed) was considered as statistically significant.

## Results

**Figure 1:**
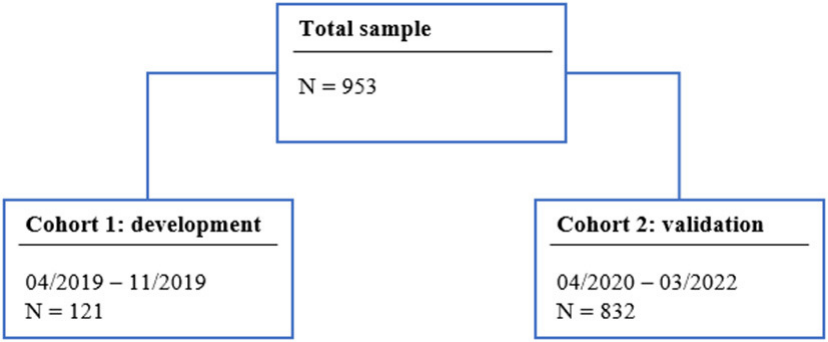
Flow-chart. N, Number.

A total of 121 patients were included in Sample 1 (development cohort) and 832 patients were analyzed in Sample 2 (validation cohort). The study's flow-chart is presented in Figure 1. In Sample 1, the patients had a median age of 77 (66–82) year and 51 patients (42.4%) were female. One-hundred fourteen patients (94.2%) had suffered an ischemic stroke and 8 (6.6%) a recurrent stroke. A median NIHSS of 1 (1–7) was recorded. In Sample 2, the median age was 73 (61–82) year, 427 (56.7%) were female, 743 (89.3%) had suffered an ischemic stroke and 126 (15.1%) a recurrent stroke. The median NIHSS amounted 3 (1–6).

Patients' median length of hospital stay was 6 (4–8) and 6 (4–9) days in Sample 1 and 2, respectively. In Sample 1, ~36% of the patients were directly discharged home, in Sample 2 this percentage was ~42%. Taking together both samples, 80 (20.6%) out of the 389 patients discharged home were living alone after stroke and 309 (79.4%) with family. A more detailed description of both samples is provided in Table 1.

**Table 1:** Patients' characteristics.

**Characteristic**	**Development cohort** **(*N* = 121)**	***N* (%)** **missing data**	**Validation cohort** **(*N* = 832)**	***N* (%)** ** missing data**	***P*-value** ** Between** **groups**
Age, years^†^	77 (66–82)	0 (0)	73 (61–82)	0 (0)	*0.0420*
Sex, female/male^‡^	51 (42.2)/70 (57.9)	0 (0)	472 (56.7)/360 (43.3)	0 (0)	*0.0036*
Stroke type, ischemic/haemorrhagic^‡^	114 (94.2)/7 (6.8)	0 (0)	743 (89.3)/89 (10.7)	0 (0)	0.1296
Recurrent stroke, yes/no^‡^	8 (6.6)/113 (93.4)	0 (0)	126 (15.1)/706 (84.9)	0 (0)	*0.0172*
Thrombolysis, yes/no^‡^	23 (19)/98 (81)	0 (0)	212 (25.5)/620 (74.5)	0 (0)	0.1526
Thrombectomy, yes/no^‡^	10 (8.3)/111 (91.7)	0 (0)	102 (12.3)/730 (87.7)	0 (0)	0.2610
Time between stroke onset and hospital admission^†^	0 (0–0)	0 (0)	0 (0–0)	0 (0)	0.2745
Time between stroke onset and assessment poststroke, days^†^	1 (1–2)	0 (0)	1 (1–3)	0 (0)	*0.0073*
Length of hospital stay, days^†^	6 (4–8)	0 (0)	6 (4–9)	0 (0)	0.7386
Living at home with or without support before stroke, yes/no^‡^	113 (93.4)/8 (6.6)	0 (0)	782 (94)/50 (6)	0 (0)	0.9559
**Clinical scales**
NIHSS (0–42)^†^	3 (1–7)	0 (0)	3 (1–6)	43 (5.2)	0.1602
Short-LIMOS (10–50)^†^	32 (20.42–39.08)	0 (0)	34.21 (24.38–41.94)	124 (14.9)	*0.0195*
Discharge destination		0 (0)		0 (0)	
Home, alone^‡^	11 (9.1)		69 (8.3)		
Home, with family^‡^	32 (26.5)		277 (33.3)		
Rehabilitation^‡^	61 (50.4)		384 (46.2)		
Temporal transitional care^‡^	0 (0)		3 (0.4)		
Other acute hospital^‡^	0 (0)		4 (0.5)		
Nursing home^‡^	14 (11.6)		70 (8.4)		
Died^‡^	3 (2.5)		25 (3.0)		
Outcome		0 (0)		0 (0)	
Discharge home, yes/no^‡^	43 (35.5)/78 (64.5)		346 (41.6)/486 (58.4)		0.2436

^†^Median (1st and 3rd quartiles).

^‡^*N* (%); Short-LIMOS, Short Version of the Lucerne ICF-Based Multidisciplinary Observation Scale; N, Number; NIHSS, National Institutes of Health Stroke Scale.

A statistical comparison of both samples showed that patients in Sample 1 were significantly older, were less often female, had more often suffered a first-ever stroke, were assessed earlier poststroke, and had a slightly lower Short-LIMOS score. A full overview of the *p*-values for all comparisons can be found in Table 1.

**Figure 2:**
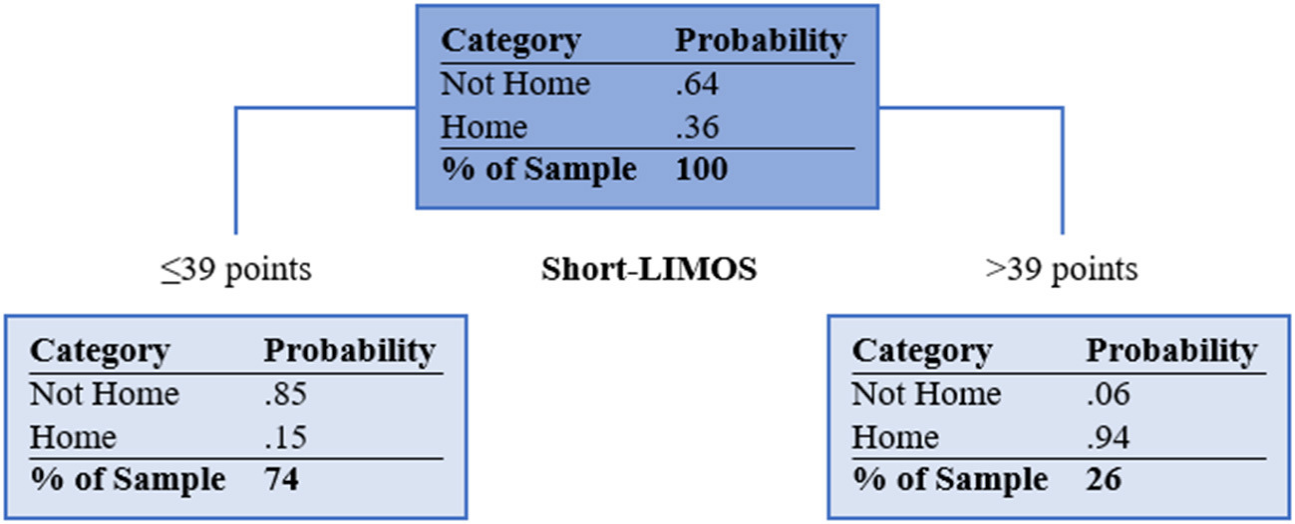
Pruned classification and regression tree. Light blue filled boxes represent the terminal nodes. Short-LIMOS, Short Version of the Lucerne ICF-Based Multidisciplinary Observation Scale. In case of missing Short-LIMOS data, age was used as a surrogate marker (<55.5 for discharge home vs. ≥55.5 years for no discharge home).

Supplementary Figure 1 shows the unpruned CART analysis. The final tree after pruning has one split and is visualized in Figure 2. The best splitting criterion for discharge home was found to be the Short-LIMOS score with a cut-off of 39/50 points. Patients with a Short-LIMOS score of ≤ 39/50 had a probability of 0.15 to be discharged home and patients with a Short-LIMOS score of >39/50 had a probability of 0.94 to be discharged home. The best surrogate split was age (<55.5 for discharge home vs. ≥55.5 years for no discharge home). The decision tree's AUC (95% confidence interval) was 0.84 (0.76, 0.91) in the development sample and 0.74 (0.72, 0.77) in the validation sample. An overview of all performance measures is presented in Table 2.

**Table 2:** Discrimination of the decision tree in the development and validation cohorts.

**Decision tree performance**	**Development cohort**	**Validation** **cohort**
	**(*N* = 121)**	**(*N* = 832)**
Accuracy (95% CI)	0.88 (0.80, 0.93)	0.77 (0.74, 0.80)
Sensitivity	0.70 (0.54, 0.83)	0.59 (0.54, 0.64)
Specificity	0.97 (0.91, 1.00)	0.90 (0.87, 0.92)
Positive predictive value	0.94 (0.79, 0.99)	0.81 (0.75, 0.85)
Negative predictive value	0.85 (0.76, 0.92)	0.75 (0.72, 0.79)
AUC (95% CI)	0.84 (0.76, 0.91)	0.74 (0.72, 0.77)

Acc, Accuracy; AUC, Area Under the Receiver Operator Characteristic Curve; CI, Confidence Interval.

A comparison between patients with and without missing data on the Short-LIMOS showed that those with missing data had significantly less often received a thrombectomy, a lower NIHSS score, a shorter length of hospital stay, and were more often discharged home. The *p*-values for all between-groups comparisons can be found in Supplementary Table 1.

At 2-month follow-up, 375 out of the 389 patients who were discharged home could be contacted by telephone, and none of them was inappropriately discharged home.

## Discussion

An easy-to-apply decision tree for early and safe discharge home after an acute stroke was developed in a sample of stroke patients, and externally validated in an independent, prospective, heterogeneous sample of patients who were discharged on a median of 6 days after admission. An excellent discrimination in the development sample and an acceptable discrimination in the validation sample was achieved by only assessing the patients' performance in the ADL with the Short-LIMOS <48 h poststroke. Furthermore, none of the patients were re-hospitalized due to their stroke symptoms during the first 2 months after hospital discharge. Since patients were included regardless of the presence or absence of common exclusion criteria (e.g., presence of prior strokes, comorbidities, prestroke dependence in the ADL, and inability to follow instructions), the decision tree is applicable in regular care, virtually without any restrictions.

The fact that the discriminative ability of the decision tree was somewhat lower in the validation sample as compared to the development sample is not surprising. This phenomenon is well described in the literature because of, for example, overfitting in the development cohort or a different case mix, in which the patient characteristics in the validation cohort differ from those in the development cohort (28). A different case mix was also found in our validation sample, which included younger patients, more females, and more recurrent strokes than the development sample. Furthermore, patients in the validation sample were assessed at a slightly later timepoint poststroke and had a marginally higher Short-LIMOS score. Despite these differences, the AUC is still acceptable and, for a clinical application, the PPV and NPV are particularly important, as they inform about the probability that an individual patient will (or will not) be discharged home (29). A PPV of 0.81 in the validation cohort can be classified as good and indicates that patients with a Short-LIMOS score of >39 are mostly correctly classified as being discharged home. The NPV of 0.75 is somewhat lower, but still acceptable. The NPV represents patients with a Short-LIMOS of ≤ 39 points who were not discharged home. Since the mean length of stay in the stroke unit was 6 days, it is conceivable that some patients, who were severely impaired in their ADL performance within 48 h, further improved over time and thus could be discharged home (i.e., false negatives). In our sample, the patients with a Short-LIMOS of ≤ 39 points who were falsely classified as “no discharge home” had a median length of stay of 6 (5–8) days.

Although we hypothesized that ADL would be an essential predictor for discharge home, we were somewhat surprised that only ADL performance <48 h poststroke (in terms of motor, cognitive, and communication abilities) needs to be assessed to make an accurate prediction. This contrasts with the findings of several reviews on this topic and the Itaya model, in which also age, living situation prior to stroke, and level of neurological deficits were found to be highly predictive (1, 2, 4). Although we selected these variables as candidate predictors in our analysis, they were not retained in the final decision tree. A possible explanation could be that the patients' age, premorbid living situation, stroke type, and neurological deficits could already *per se* impact their actual ADL performance (i.e., a negative effect of higher age, not living at home, having suffered a hemorrhagic stroke, and more severe neurological deficits would be already included and reflected by lower Short-LIMOS scores). A *post-hoc* analysis showed indeed a moderate negative correlation between age and the Short-LIMOS in our sample (rs −0.36, *p* < 0.0001). Furthermore, patients with a hemorrhagic stroke had significantly lower Short-LIMOS scores than those with an ischemic stroke [median 26.08 (14.83–35.04) vs. 34.75 (24.67–42), *p* < 0.0001], and patients not living at home prior to their stroke had significantly lower Short-LIMOS scores than those who did live at home [median 19.17 (13.33–26.83) vs. 34.71 (24.92–42), *p* < 0.0001]. When considering how ADLs were measured, a comparison of our decision tree with the Itaya model shows that the motor part of the Functional Independence Measure had more weight than its cognitive counterpart (4). Contrary to this, the Short-LIMOS in our decision tree has an equal weighting between—on the one hand—the patients' cognitive and communication abilities and—on the other hand—their motor capabilities. In addition, the Short-LIMOS assessment shows no floor or ceiling effects, because it includes cognitive, communication, and motor tasks of both higher and lower-order complexity (16). These could be further reasons as to why only the Short-LIMOS score was retained in the final classification and regression tree.

The Functional Independence Measure (30) and the (extended) Barthel Index (31) are two well-established measures for assessing stroke patients' ADL performance, whereas the Short-LIMOS is a new assessment tool. Nevertheless, we advocate the use of the Short-LIMOS at acute stroke units, as the instrument is short and compact. Moreover, due to its multidisciplinary nature, the Short-LIMOS allows to gain a more comprehensive picture of the patient's ADL performance than with both the above-mentioned, established scales (16). In particular, the observation of patients' performance in complex tasks, as well as the patients' cognitive and communication abilities (16), are of central importance, as these are typically a prerequisite for an independent functioning at home.

The strengths of the present study reside in its wide inclusion criteria, allowing a broad application of the decision tree in clinical practice, in the prospective nature of data collection in the clinical routine, in the presence of both a development and a validation cohort that were separated by an interval of time [instead of using a split sample method to define two samples (20)], and in its adequate sample size. A potential limitation is the moderate percentage of missing data on the main predictor (Short-LIMOS) in the validation cohort (~15%). An advantage of the applied CART analysis is that surrogate markers are automatically used when data on the retained predictor variables are missing. In case of missing data on the Short-LIMOS, age (<55.5 vs. ≥55.5 years) was used as a surrogate marker and thus also patients with missing Short-LIMOS data could be included in our analysis. The advantage is that this reflects clinical practice—as also there, some data may be missing—and the results are not biased due to a complete-case analysis. It should, however, be noted that this surrogate marker is less good in splitting into homogeneous groups than the Short-LIMOS itself. With that, our external validation is rather conservative and does not overestimate the decision tree's performance. Closer inspection of the patient characteristics showed that patients with missing Short-LIMOS data were more often treated with a mechanical thrombectomy, showed less neurological deficits, had a shorter length of hospital stay, and were discharged home more often when compared to patients without missing data.

To increase the generalizability of the presented decision tree, a next step would be to test its external validity in other countries with a shorter length of hospital stay and a different organization of health care. Furthermore, the decision tree's impact on clinical decision making, patient's outcome, length of hospital stay, and costs should be investigated (28). Although a cluster randomized trial is considered to be the best study design for testing a prediction model's impact, such a design is time-consuming and expensive (28). As an alternative, a pre-post design could be used (28, 32). In the first observational period, the model is not presented to clinicians and data on outcomes are collected. In the next step, the model is introduced and the outcomes within this second stage are compared with those of the first stage.

## Conclusion

To summarize, in stroke patients who have mild limitations in ADL performance <48 h after stroke onset, the decision tree developed in the present study is good in predicting early and safe discharge home. In patients with moderate to severe limitations in the ADLs, the prediction concerning not being discharged home is fair. Importantly, patients discharged home did not need readmission to the hospital or to a rehabilitation center due to an improper decision on discharge destination. As this tree was externally validated in stroke patients regardless of their pre-stroke disability and comorbidity, as well as the number of strokes, it has the potential to find broad application in settings in which the length of hospital stay after an acute stroke is short (<1 week).

## Data availability statement

The original contributions presented in the study are included in the article/Supplementary material, further inquiries can be directed to the corresponding author.

## Ethics statement

The studies involving human participants were reviewed and approved by Cantonal Ethics Committee Northwest and Central Switzerland. The patients/participants provided their written informed consent to participate in this study.

## Author contributions

JV, BO, TV, and TN conceptualized the study and determined the study's methodology. TN gained ethical approval. Data curation was done by BO. The formal analysis was performed by JV. The results were interpreted by all authors. JV wrote the original draft. All authors reviewed and edited the manuscript and approved the final version of the manuscript.

## Funding

This study was supported by the Swiss National Science Foundation (Grant No. 32003B_196915). The funder played no role in the research.

## Conflict of interest

The authors declare that the research was conducted in the absence of any commercial or financial relationships that could be construed as a potential conflict of interest.

## Publisher's note

All claims expressed in this article are solely those of the authors and do not necessarily represent those of their affiliated organizations, or those of the publisher, the editors and the reviewers. Any product that may be evaluated in this article, or claim that may be made by its manufacturer, is not guaranteed or endorsed by the publisher.
