# Supplementary material for: Speed up discharge planning at the acute stroke unit: A development and external validation study for the early prediction of discharge home
Source: Front Neurol. 2022 Sep 16;13:999595. doi: 10.3389/fneur.2022.999595 (PMC9523004; doi:10.3389/fneur.2022.999595)
Supplement: Supplementary file 3 [file Data_Sheet_1.docx]

Supplementary Material

Speed up discharge planning at the acute stroke unit: a development and external validation study for the early prediction of discharge home

Janne Marieke Veerbeek^1*^, Beatrice Ottiger^1^, Dario Cazzoli^1,2,3^, Tim Vanbellingen^1,2^, Thomas Nyffeler^1,2,4^

^1^Neurocenter, Luzerner Kantonsspital, Lucerne, Switzerland

^2^ARTORG Center for Biomedical Engineering Research Gerontechnology & Rehabilitation Group, University of Bern, Bern, Switzerland

^3^Department of Psychology, University of Bern, Bern, Switzerland

^4^Department of Neurology, Inselspital, Bern University Hospital, University of Bern, Switzerland

*** Correspondence:**Janne M. Veerbeek
janne.veerbeek@luks.ch

# Short - Lucerne ICF-Based Multidisciplinary Observation Scale (Short-LIMOS)

## Manual


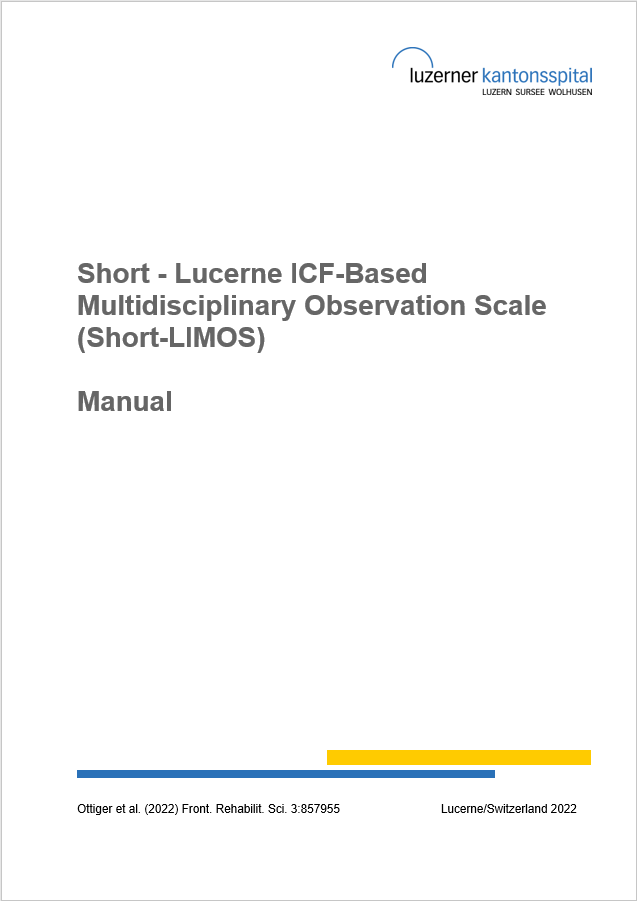


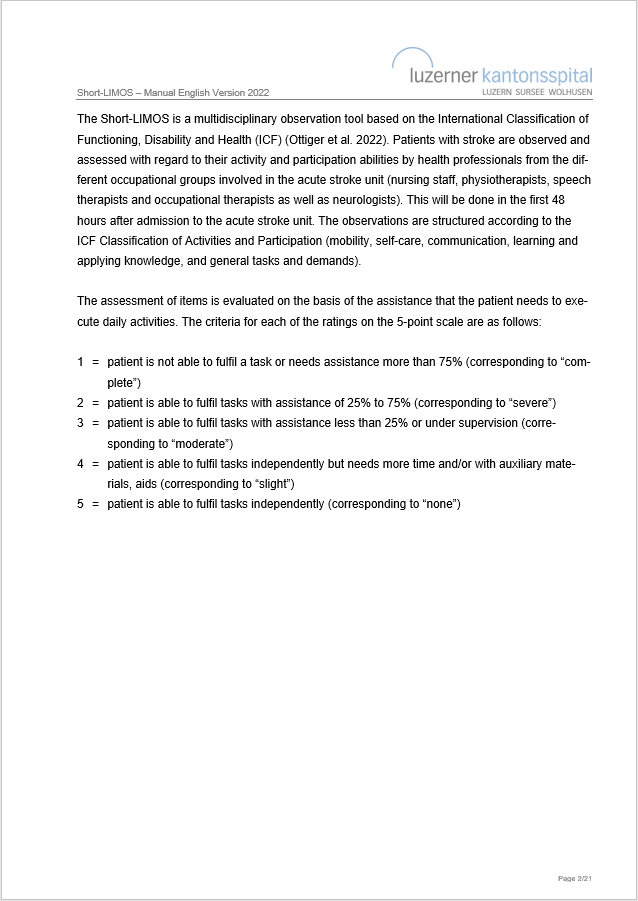


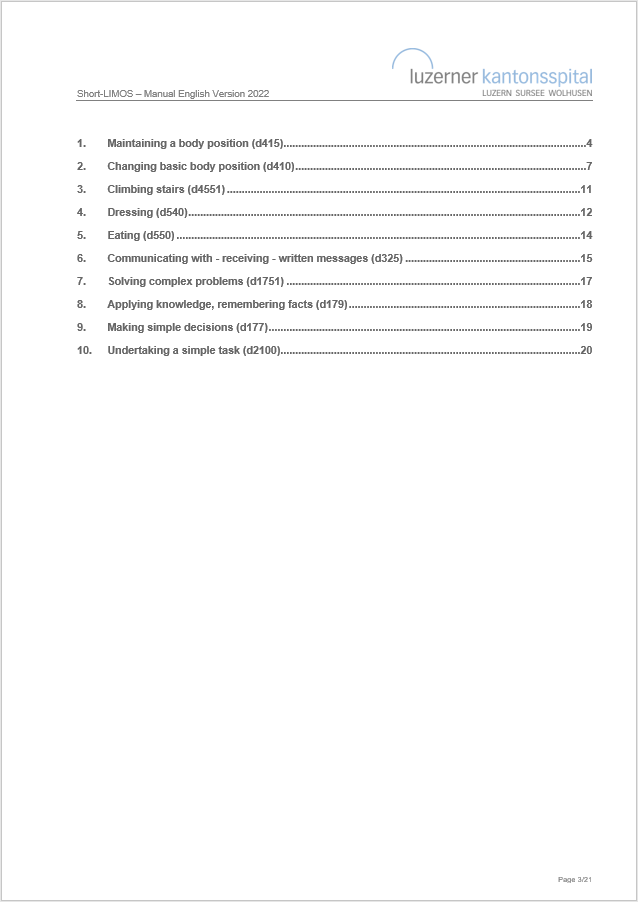


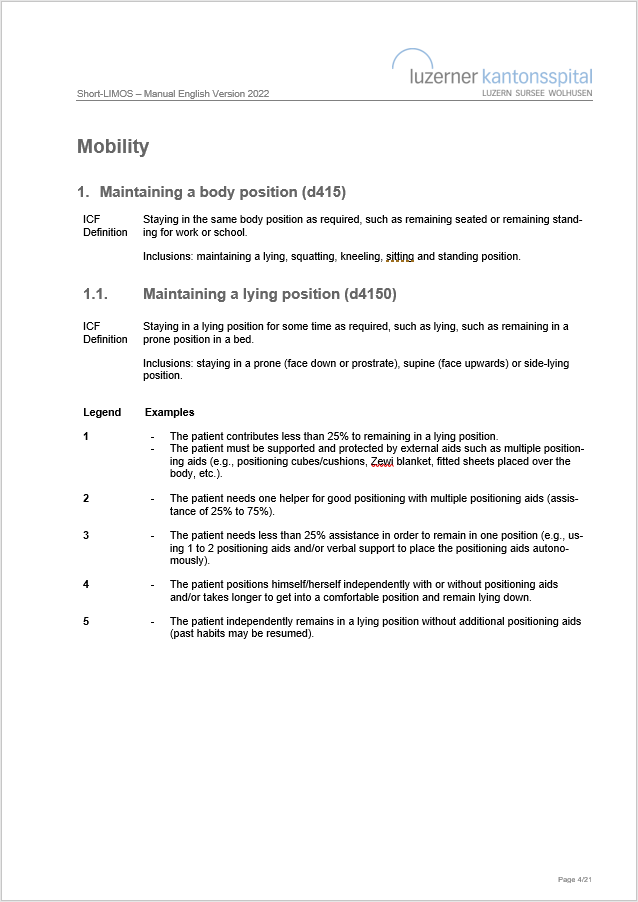


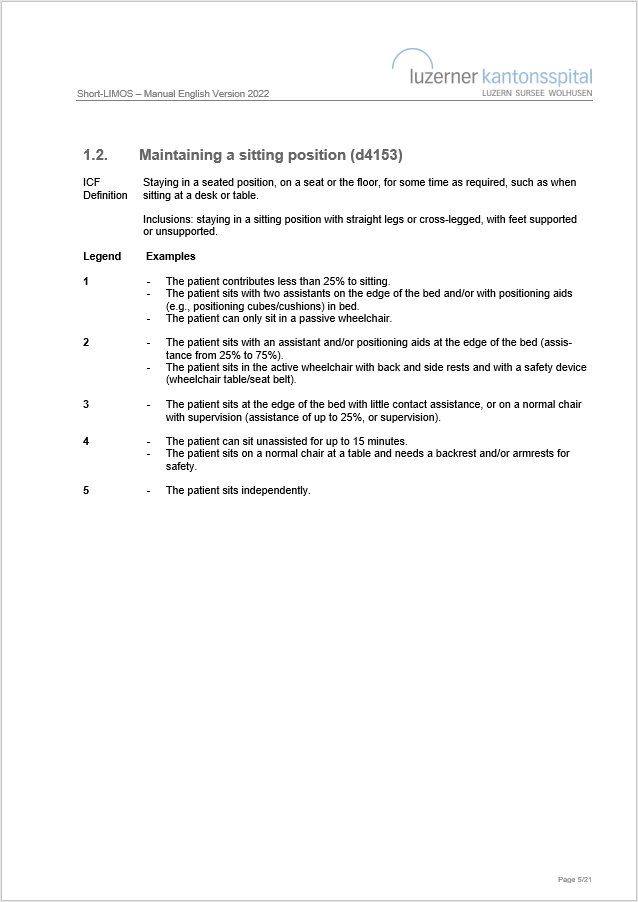


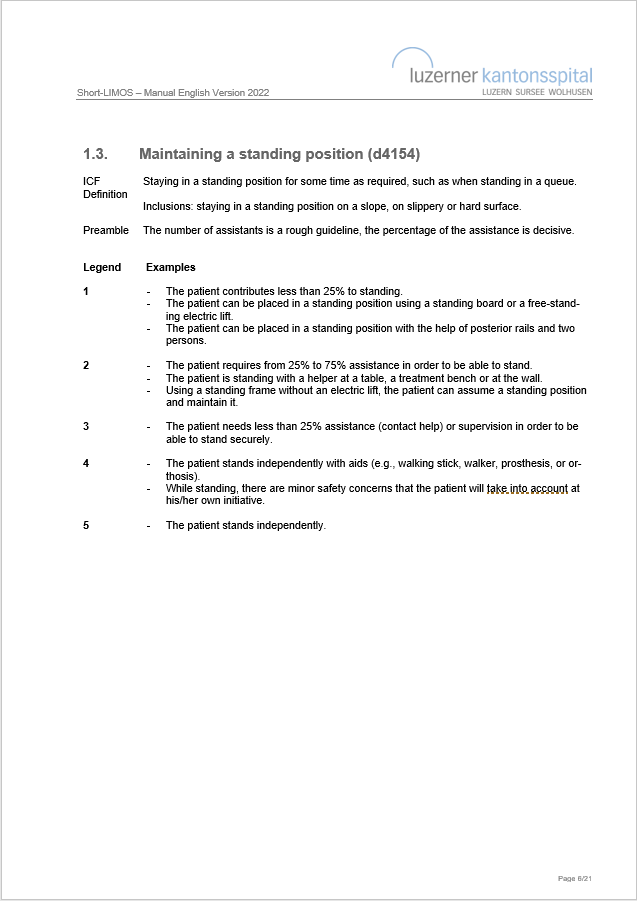


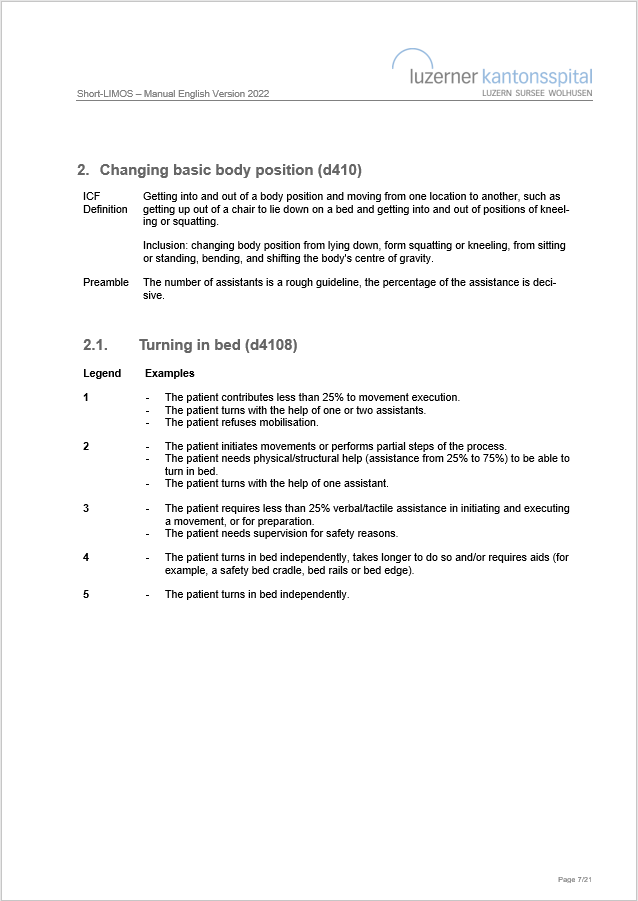


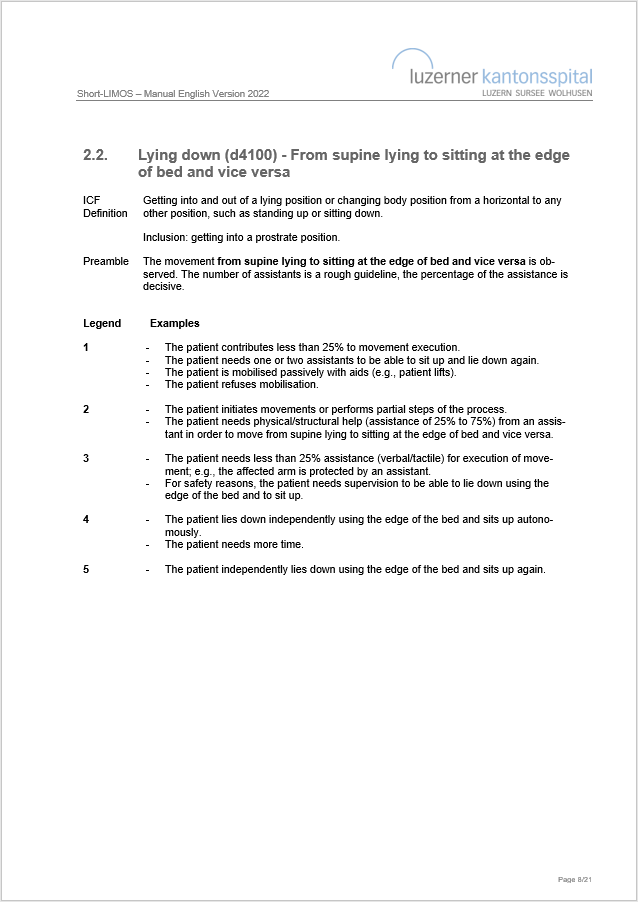


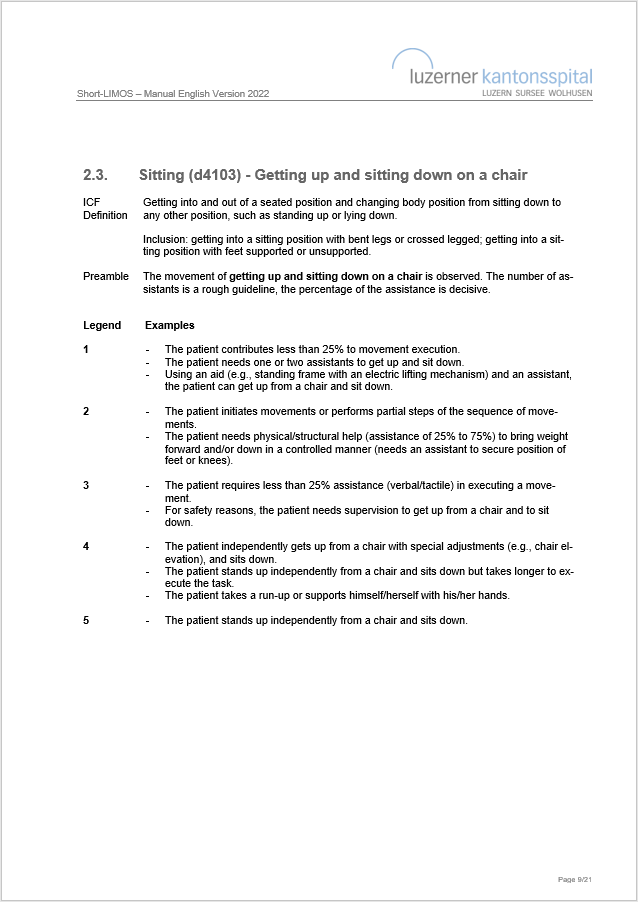


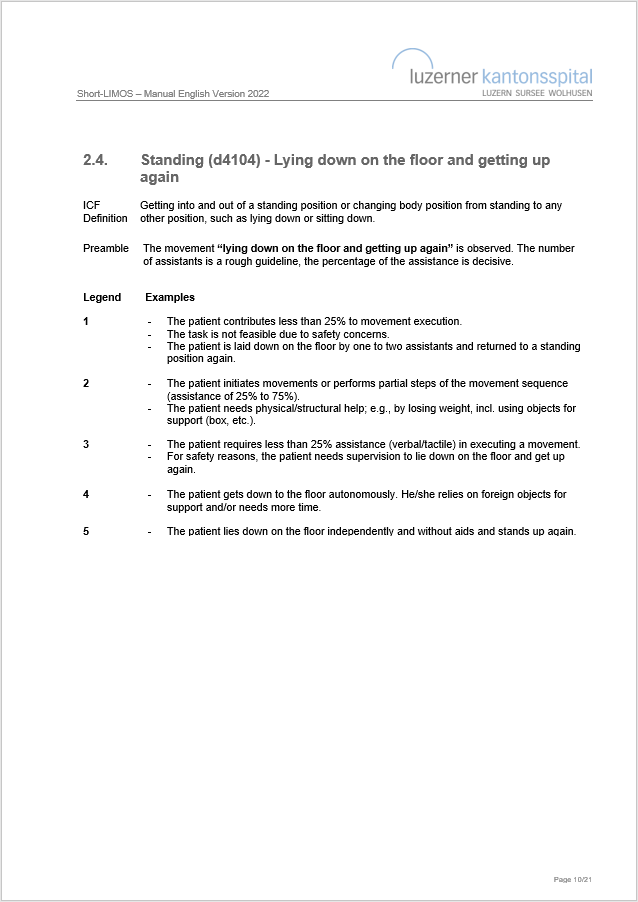


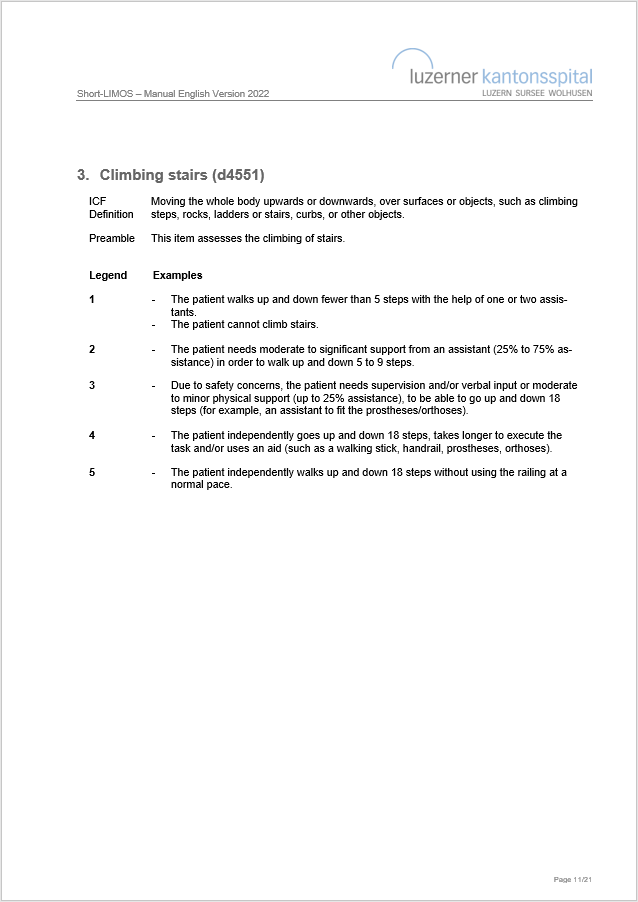


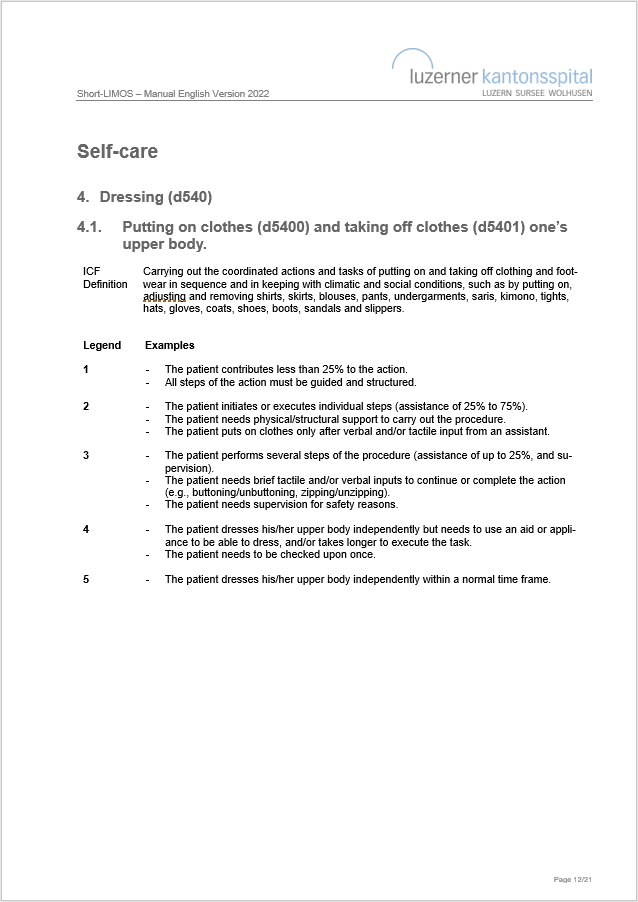


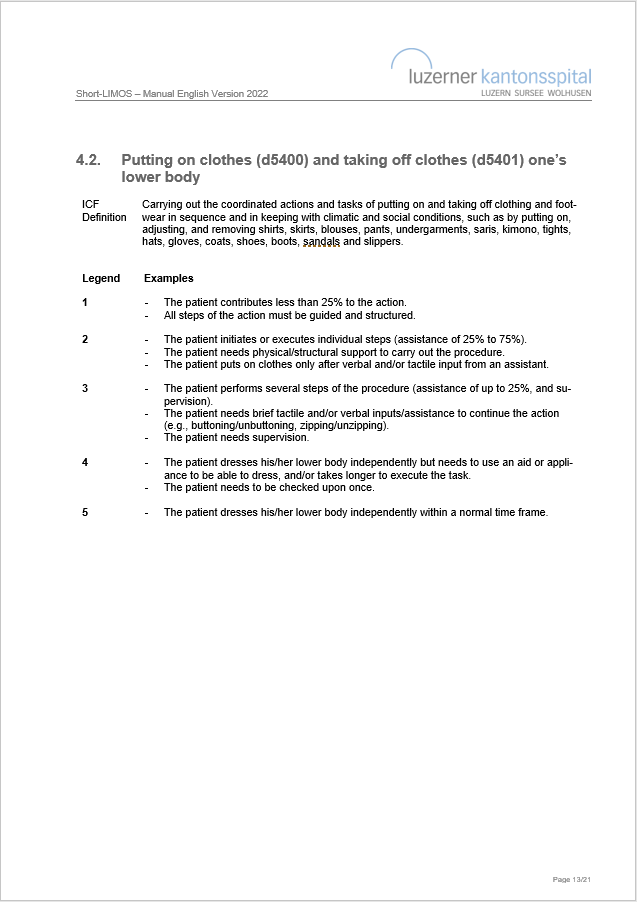


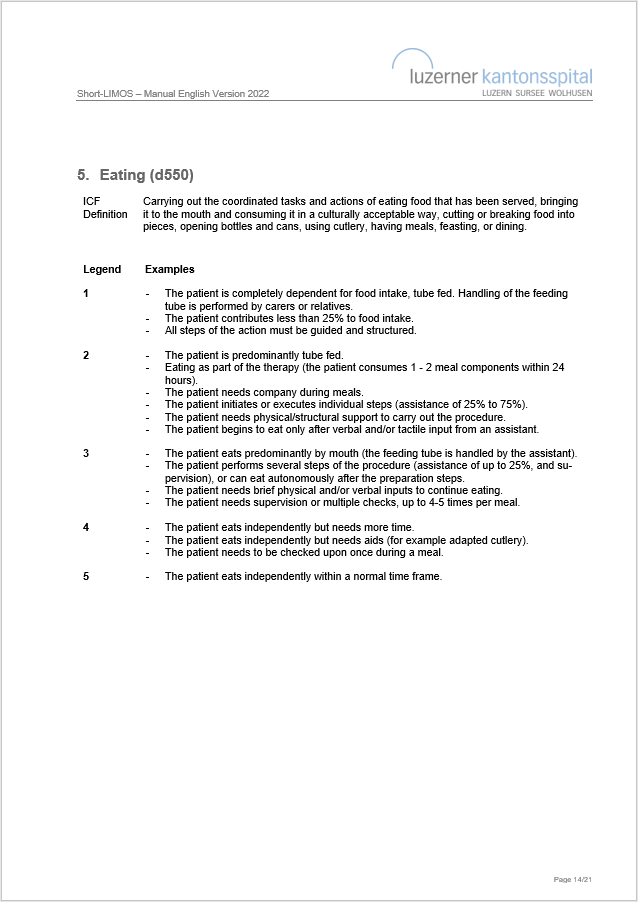


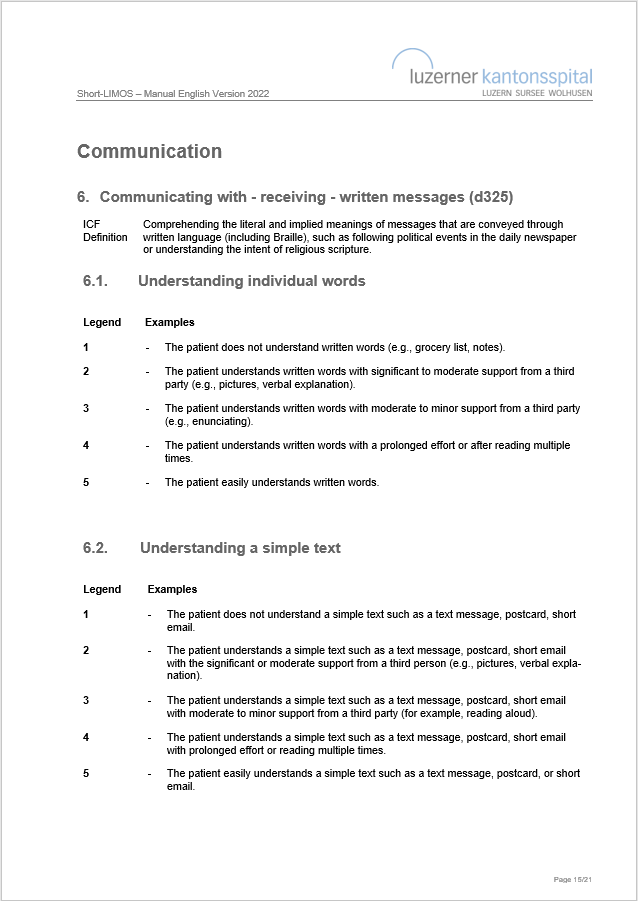

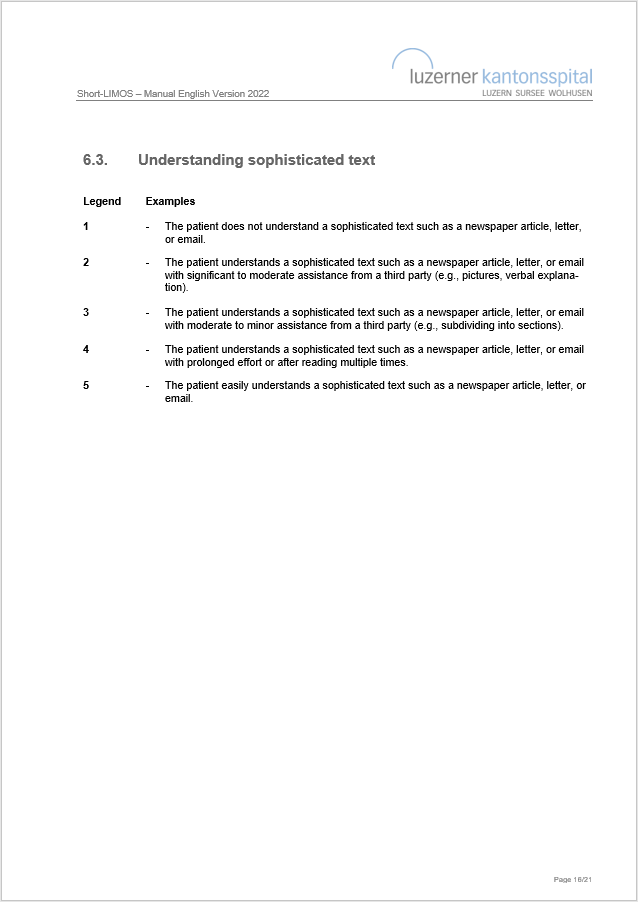

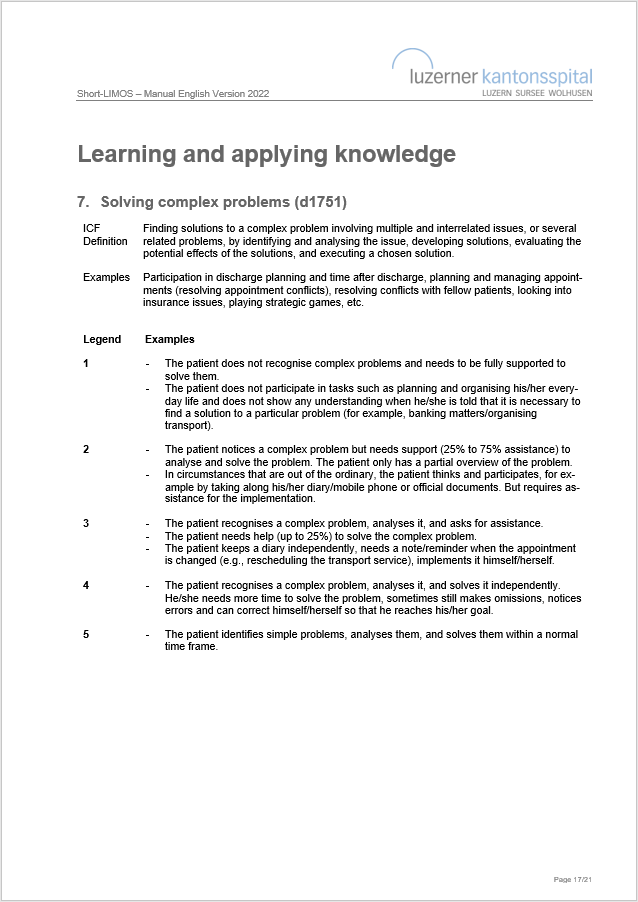

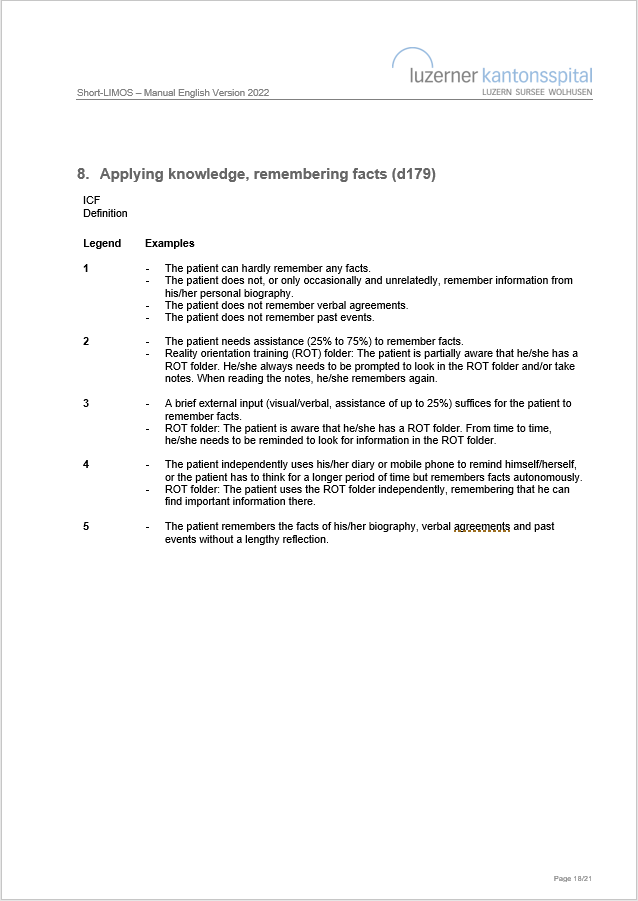

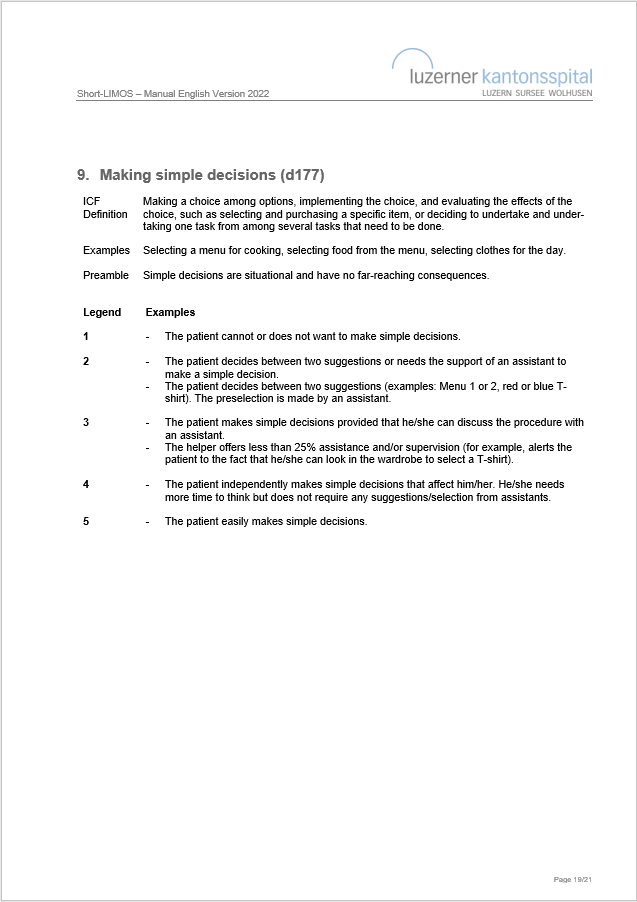

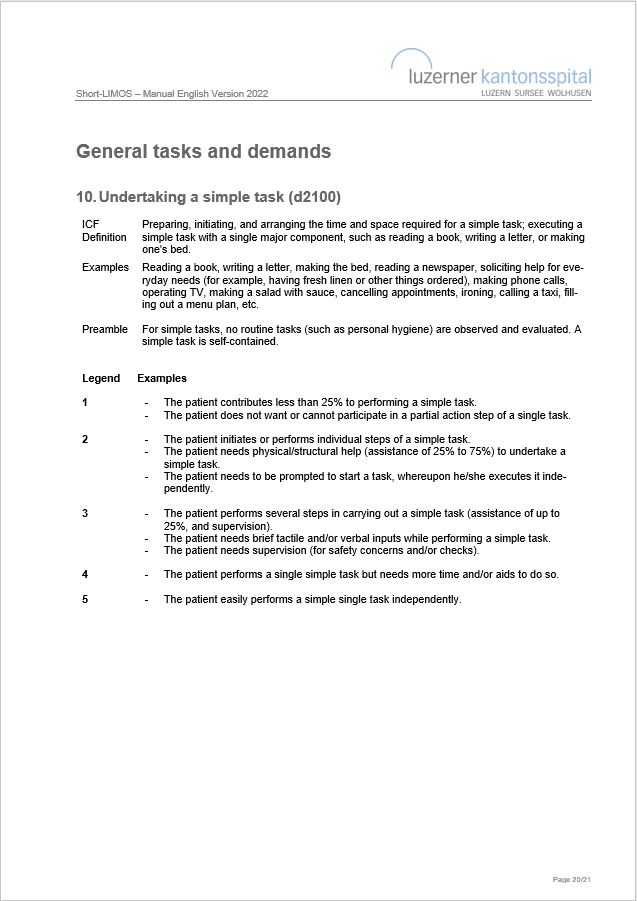

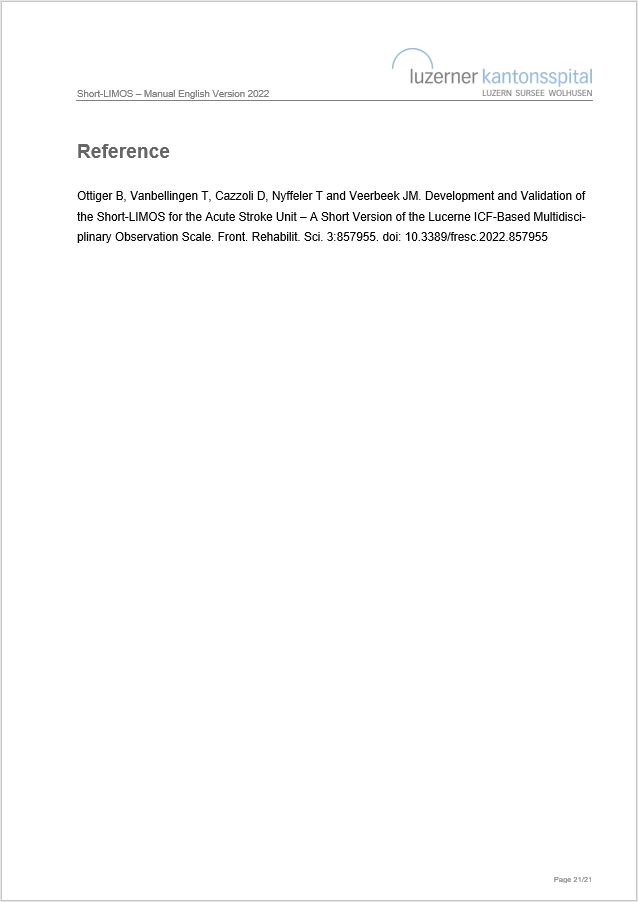


## Form


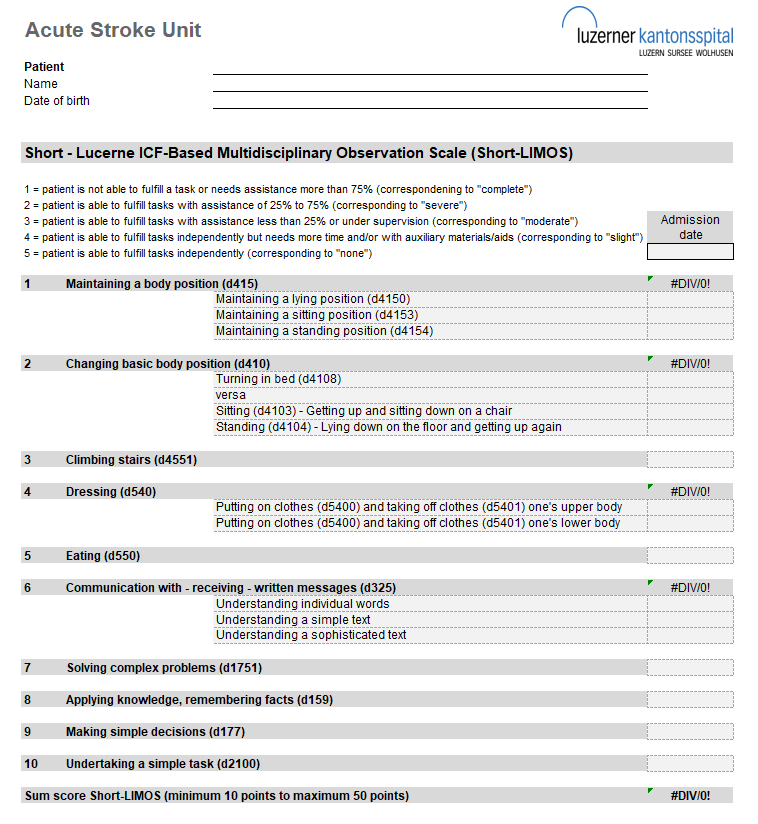


Ottiger B, Vanbellingen T, Cazzoli D, Nyffeler T, Veerbeek JM. Development and validation of the Short-LIMOS for the acute stroke unit - A short version of the Lucerne ICF-based Multidisciplinary Observation Scale. Front Rehabil Sci. (2022) 3:857955. doi: 10.3389/fresc.2022.857955

# Supplementary Figures and Tables

## Supplementary Table

**Supplementary Table 1.** Comparison of patients with and without missing Short-LIMOS data in Sample 2

| **Characteristic** | **Patients with missing data**  (N=124) | **Patients without missing data**  (N=708) | **P-value** Between groups |
| --- | --- | --- | --- |
| Age, years † | 73 (64.75–82) | 73 (61–82) | 0.4147 |
| Sex, female/ male ‡ | 72 (58.1)/ 52 (41.9) | 400 (56.5)/ 308 (43.5) | 0.8206 |
| Stroke type, ischemic/ hemorrhagic ‡ | 115 (92.7)/ 9 (7.3) | 628 (88.7)/ 80 (11.3) | 0.2357 |
| Recurrent stroke, yes/ no ‡ | 26 (21)/ 98 (79) | 100 (14.1)/ 608 (85.9) | 0.0680 |
| Thrombolysis, yes/ no ‡ | 25 (20.2)/ 99 (79.8) | 187 (26.4)/ 521 (73.6) | 0.1732 |
| Thrombectomy, yes/ no ‡ | 8 (6.5)/ 116 (93.5) | 94 (13.3)/ 614 (86.7) | *0.0467* |
| Time between stroke onset and hospital admission, days † | 0 (0–0) | 0 (0–0) | 0.6265 |
| Time between stroke onset and assessment, days † | 1 (1–3) | 1 (1–3) | 0.9792 |
| Length of hospital stay, days † | 4 (3–6) | 6 (4–10) | *<0.0001* |
| Living at home with or without support before stroke, yes/ no ‡ | 115 (92.7)/ 9 (7.3) | 667 (94.2)/ 41 (5.8) | 0.6677 |
| Clinical scale |  |  |  |
| NIHSS (0–42) † | 2 (0–4) | 3 (1–7) | *<0.0001* |
| Discharge destination |  |  | *0.0002* |
| Home, alone ‡ | 12 (9.7) | 57 (8.1) |  |
| Home, with family ‡ | 62 (50) | 215 (30.4) |  |
| Rehabilitation ‡ | 34 (27.4) | 350 (49.4) |  |
| Temporal transitional care ‡ | 0 (0) | 3 (0.4) |  |
| Other acute hospital ‡ | 1 (0.8) | 3 (0.4) |  |
| Nursing home ‡ | 9 (7.3) | 61 (8.6) |  |
| Died ‡ | 6 (4.8) | 19 (2.7) |  |
| Outcome |  |  |  |
| Discharge home, yes/ no ‡ | 74 (59.7)/ 50 (40.3) | 272 (38.4)/ 436 (61.6) | *<0.0001* |

Legend: †, Median (1^st^ and 3^rd^ quartiles); ‡, N (%); Short-LIMOS, Short Version of the Lucerne ICF-Based Multidisciplinary Observation Scale; N, Number; N, Number; NIHSS, National Institutes of Health Stroke Scale.

## Supplementary Figure

**Supplementary Figure 1.** Unpruned classification and regression tree


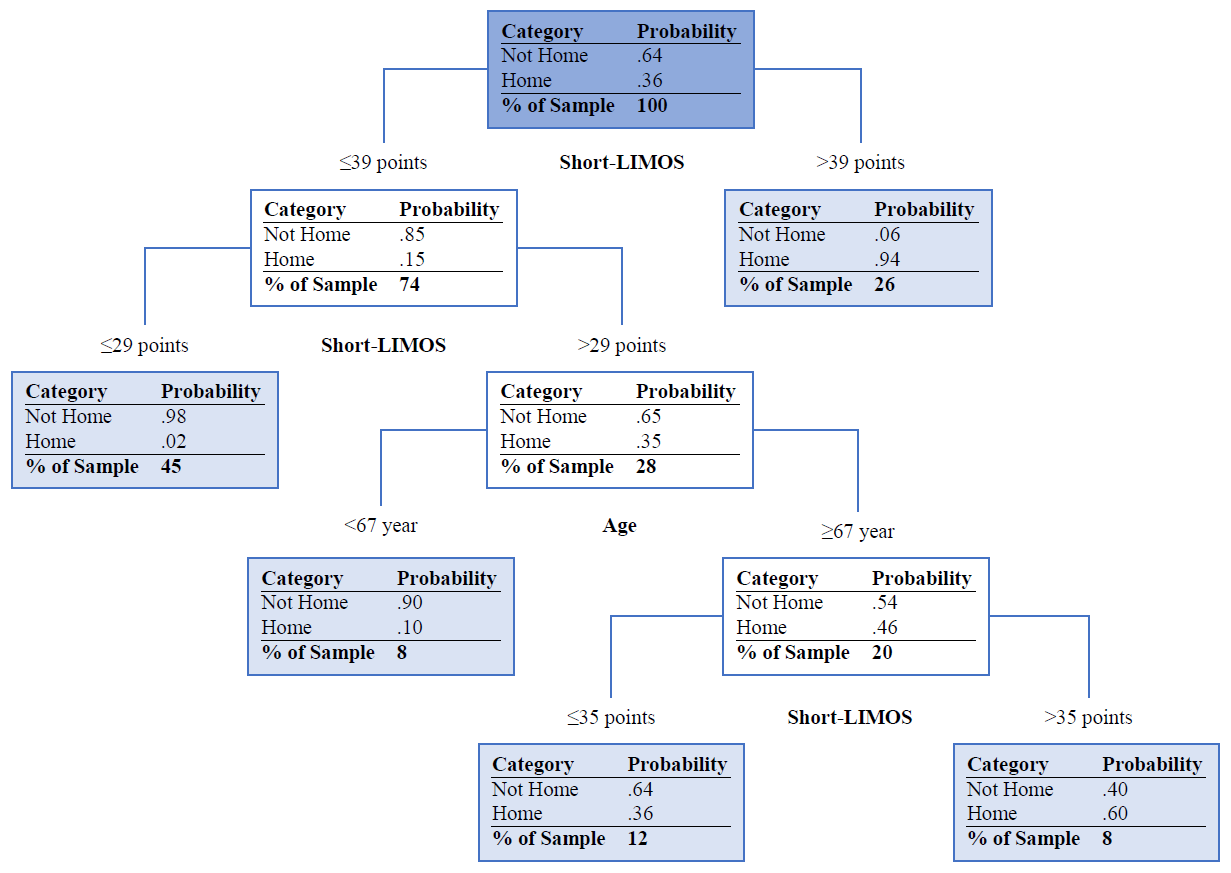
Legend: Light blue filled boxes represent the terminal nodes.
